# Fluorescence Correlation Spectroscopy Reveals Interaction of Some Microdomain-Associated Lipids with Cellular Focal Adhesion Sites

**DOI:** 10.3390/ijms21218149

**Published:** 2020-10-31

**Authors:** Christian Kleusch, Cornelia Monzel, Krishna Chander Sridhar, Bernd Hoffmann, Agnes Csiszár, Rudolf Merkel

**Affiliations:** 1 Institute of Biological Information Processing, IBI-2: Mechanobiology, Forschungszentrum Jülich GmbH, 52425 Jülich, Germany; Christian.Kleusch@nanotempertech.com (C.K.); cornelia.monzel@hhu.de (C.M.); k.sridhar@fz-juelich.de (K.C.S.); b.hoffmann@fz-juelich.de (B.H.); a.csiszar@fz-juelich.de (A.C.); 2 Experimental Medical Physics, Department of Physics, Heinrich-Heine Universität Düsseldorf, 40225 Düsseldorf, Germany

**Keywords:** lipid diffusion, focal adhesion site, fluorescence correlation spectroscopy (FCS), membrane microdomains, model membrane, liquid ordered and disordered phases, fusogenic liposomes

## Abstract

Cells adhere to the extracellular matrix at distinct anchoring points, mostly focal adhesions. These are rich in immobile transmembrane- and cytoskeletal-associated proteins, some of which are known to interact with lipids of the plasma membrane. To investigate their effect on lipid mobility and molecular interactions, fluorescently labeled lipids were incorporated into the plasma membranes of primary myofibroblasts using fusogenic liposomes. With fluorescence correlation spectroscopy, we tested mobilities of labeled microdomain-associated lipids such as sphingomyelin (SM), ganglioside (GM1), and cholesterol as well as of a microdomain-excluded phospholipid (PC) and a lipid-like molecule (DiIC_18_(7)) in focal adhesions (FAs) and in neighboring non-adherent membrane areas. We found significantly slower diffusion of SM and GM1 inside FAs but no effect on cholesterol, PC, and DiIC_18_(7). These data were compared to the molecular behavior in L_o_/L_d_-phase separated giant unilamellar vesicles, which served as a model system for microdomain containing lipid membranes. In contrast to the model system, lipid mobility changes in FAs were molecularly selective, and no particle enrichment occurred. Our findings suggest that lipid behavior in FAs cannot be described by L_o_/L_d_-phase separation. The observed slow-down of some molecules in FAs is potentially due to transient binding between lipids and some molecular constituent(s).

## 1. Introduction

Adhesion of mammalian cells to the extracellular matrix is decisive for physiological and pathological processes such as tissue maintenance and repair, cell locomotion, and scar formation. In all these processes, mechanical forces have to be transmitted across the cell membrane. As the membrane is fluid and cannot sustain static forces, transmembrane protein linkages are indispensable for this task. They are furnished by focal adhesions (FAs) that are seen in cultured cells as micron-sized, protein-rich plaques [1]. FAs have been studied intensely with correspondingly deep insight into structure [2], life cycle [3,4,5], molecular composition [6], and relation to disease [7], just to name a few aspects. The central elements of FAs are heterodimeric cell adhesion molecules of the integrin family. They are composed of one α and one β chain that span the plasma membrane and together form binding domains for molecular motifs on the extracellular matrix (ECM). The 24 different integrins known in humans cover a wide spectrum of ECM target motifs [8]. Integrins are connected to a plaque of many different adaptor molecules such as vinculin and talin that together form connections to the intracellular actin cytoskeleton [9,10,11].

The central function of FAs is to form a mechanical connection between scaffold structures at the inside of the cell to such at its outside. To this end, they span the fluid cellular plasma membrane, which is composed of a bilayer of lipids that act as solvent for membrane proteins. While this two-dimensional fluid appears to be a simple structure, a plethora of experimental evidence points towards a highly inhomogeneous lateral distribution of lipids and proteins, see for example [12,13]. How this lateral organization of lipids and proteins is brought about and how it is to be interpreted has been the focus of intense debates for the last three decades, the state of which has been recently reviewed by Nyholm [14] and Lu et al. [15]. In a nutshell, two synergistic processes overlap. First, physical interactions between membrane proteins and lipids induce local demixing of membrane lipids. Examples of such interactions are the specific binding between protein and lipid moieties [16], electrostatic interactions between negatively charged lipids and cationic amino acid residues [17,18], and the so-called mattress effect, which essentially dictates that the lengths of the hydrophobic protein and lipid domains must match in order to minimize free energy [14]. Second, lipid mixtures containing cholesterol and sphingomyelin (or other high melting lipids) together with unsaturated lipids, which were detected in FAs, have a high propensity for liquid–liquid phase separation [19,20,21].

This separation into liquid ordered (L_o_) and liquid disordered (L_d_) phases has been intensely studied in model membranes composed of a phospholipid with high melting temperature (often sphingomyelin or ganglioside), another one with low melting temperature (often a phosphatidylcholine with unsaturated chains), and cholesterol at about equal molecular amounts, because here, large domains are formed that can be easily observed [15,19,20]. As such model systems are superbly defined and thus enable detailed investigations, they have been instrumental for the understanding of the cellular biomembrane. Nevertheless, a cellular membrane contains hundreds of different lipid species, a high fraction of protein, and is far from thermal equilibrium. Therefore, transfer of results from model systems to cellular membranes requires utmost care.

We expect lipid–protein interactions to be frequent within FAs because of the high density of transmembrane integrins. For example, Wiseman et al. detected an integrin density of around 50–390 mol/µm^2^ in non-adhered plasma membrane regions, but 1.3–2 fold higher in young FAs and even six-fold higher in mature FAs [22]. In experiments on nanostructured substrates, similar densities of integrin ligands have been found to be necessary for the formation of stable adhesions [23,24]. In view of these high densities, it is surprising that a recent single molecule study found uninhibited diffusion of non-FA membrane proteins within FAs whereas β_3_ integrins underwent frequent arrests [25]. Based on this result, it appears likely that lipid molecules can rapidly exchange between the FA area and the rest of the plasma membrane, whereas lipid binding to FA components should result in reduced mobility and/or enrichment of the respective lipid.

At present, several components of FAs are known to interact strongly with lipid molecules. Integrins were reported to enrich anionic lipids at their transmembrane moieties [17,18], the tail domain of vinculin was found to insert into membranes [26], the same protein also contains a binding site for phosphatidylinositol-4,5-bisphosphate (PIP2) lipids that is relevant for vinculin activation [27,28], and finally, talin was also shown to bind PIP2 lipids [16,29,30].

Given these protein–lipid interactions, effects on lipid composition and/or mobility within FAs should arise. In line with this hypothesis are experiments with a polarity sensitive fluorescent probe where a high overall order was found in this region [31]. Beyond this, Fuentes et al. [32] reported that lipid domains enriched in the ganglioside GM1 respond heavily to mechanical deformation by domain growth and talin recruitment. While the aforementioned works seem to point towards a propensity of FAs for the L_o_ phase, plasma membrane modification with detergents [33] and model membrane work [34] indicate instead that integrins and FAs might attract the less ordered L_d_ phase. Many more details on the interplay of integrin-based cell adhesions and membrane microdomains can be found in a recent review by Lietha and Izard, who also point out that many aspects of this interplay are still not understood [35].

So far, direct measurements of lipid mobility together with signatures of a preferential lipid phase enrichment in FAs are missing, wherefore we here set out to explore lipid diffusion in situ. We measured lipids commonly associated with L_o_ or L_d_ phases within and outside of FAs and compared their number and mobility changes to the behavior in the well-controlled environment of model membranes. Using fluorescence correlation spectroscopy (FCS), the enrichment of particular lipids within FAs should be detectable via an increase in molecular counts. At the same time, lipid mobility changes were determined in model membranes and compared to the relative changes in FAs in cells. The combined analyses of L_o_ or L_d_ phase associated lipids then provided insights into the lipid membrane organization in FAs. To our knowledge, no direct measurement of lipid enrichment and mobility changes in FAs have been reported before.

To facilitate these measurements, we used myofibroblasts that had been differentiated from primary cardiac fibroblasts [36]. These cells exhibit very large, so-called supermature FAs containing large amounts of α_5_β_3_ and in some instances also α_5_β_1_ integrins [37,38]. Fluorescently labeled analogs of the lipids sphingomyelin (SM), ganglioside GM1, cholesterol, and phosphatidylcholine as well as the membrane probe DiIC_18_(7) were incorporated into plasma membranes of such cells, and their diffusion was measured inside and next to FAs. For comparison, the same lipid probes were investigated in phase-separated model membranes. While SM, GM1, and cholesterol were shown to have a preference for the L_o_ phase and phosphatidylcholine as well as DiIC18(7) for the L_d_ phase [39,40,41], the fluorescent counterparts of SM, GM1, and cholesterol could exhibit different behavior [42,43]. For this reason, the model membrane served also to verify the behavior of the fluorescent molecules during phase separation. As will be detailed below, we found that diffusivities of SM and GM1 were slowed in FAs, whereas this was not the case for cholesterol and phospholipid (PC).

## 2. Results

### 2.1. Localization of Focal Adhesion Sites of Cardiac Myofibroblasts

**Figure 1:**
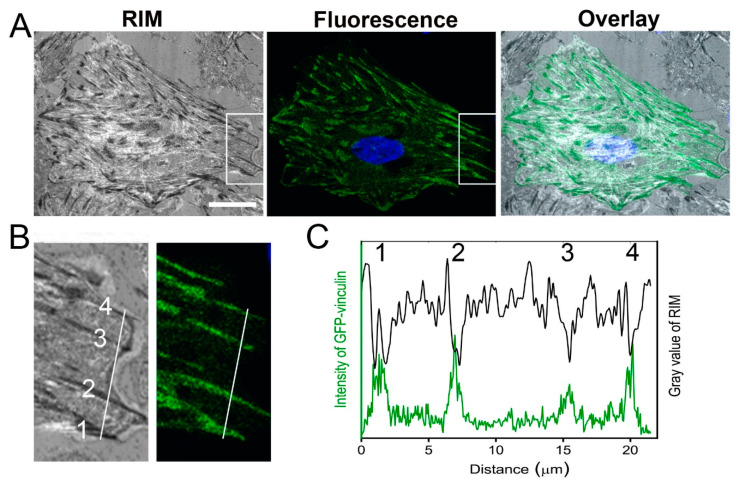
Identification of focal adhesion sites. (**A**) Localization of focal adhesions (FAs) of cardiac myofibroblasts using reflection interference microscopy (RIM) (left) and fluorescence microscopy (middle, green fluorescent protein (GFP)-vinculin–green, NucBlue–blue). The dark spots in reflection and the intense fluorescent spots of GFP-vinculin are widely co-localized (overlay is shown on the right). Scale bar, 20 µm. (**B**) Detail of marked areas in (**A**) and (**C**) overlay of intensity profiles of FAs (1–4) along marked line.

As was verified by immunostaining (see Material and Methods part and Figure S1 in the Supplementary Material) cardiac myofibroblasts could be clearly identified within the mixed co-culture using phase contrast microscopy based on their larger size and rounder shape when compared to myocytes. Myocytes were identified by co-localization of actin-cytoskeleton and α-actinin networks. These cells were smaller than fibroblasts and were spread out in a sail-like fashion, whereas fibroblasts had a rounder shape. Analyzing FAs, reflection interference microscopy (RIM) clearly showed many dark streaks (0.5–2 µm width, 1–6 µm length) all over the cell body (Figure 1A). These structures are typical for focal adhesions [1]. This assignment was verified by imaging cardiac myofibroblasts transfected with green fluorescent protein (GFP)-vinculin (Figure 1A), which is a well-established and reliable marker protein of focal adhesions [44]. Some of those focal adhesions are shown in detail in Figure 1B, their intensity profile in both channels are plotted in Figure 1C. Our results clearly confirm previous findings [1,45,46], therefore in the following, only FAs that could be unambiguously identified in RIM were analyzed.

### 2.2. Distribution of Fluorescent Membrane Components in the Plasma Membrane of Cardiac Myofibroblasts

For measurement of lipid diffusion, fluorescently labeled molecules were intercalated into the plasma membrane of cardiac myofibroblasts using fusogenic liposomes. For the sake of consistency, molecules labeled with the same fluorescent dye at equivalent locations (the alkyl-chain region) and the well-studied synthetic fluorescent probe DiIC_18_(7) were chosen. Since lipids chemically modified at their polar head groups or at their alcyl-chains, many exhibit different phase separation behavior compared to their unlabeled counterparts [42,43], and since the polar head groups are often involved in molecular binding, e.g., via hydrogen bonds, alkyl-chain modified lipids were chosen. We used model membranes from a lipid mixture that is well known to phase separate into L_o_ and L_d_ phases [20] to identify the fluorescent probe behavior and used this as a reference for data interpretation in FAs. As fluorescent derivatives of lipids whose unlabeled versions are associated with the L_o_ phase, we used BODIPY FL-sphingomyelin (BFL-SM), BODIPY FL-ganglioside (BFL-GM1), and TopFluor-cholesterol (TopChol), also a BODIPY derivative, whereas β-BODIPY-C_12_HPC (BFL-PC) was used as reporter for the L_d_ specific phosphatidylcholine. For molecular structures, see Figure S2. In addition, we analyzed the diffusion of DiIC_18_(7), a synthetic dialkylcarbocyanine membrane probe without natural analog. In cells, it serves as a non-bioactive reference membrane probe and in model membranes as a selective marker for the liquid disordered lipid phase (L_d_) similar to other carbocyanine dyes [34,45].

**Figure 2:**
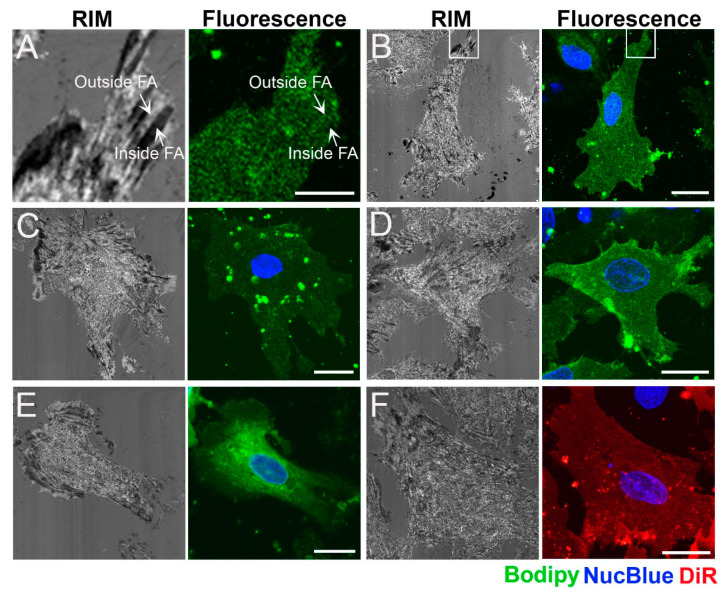
Primary cardiac fibroblasts with maturated focal adhesion sides on the lamellipodia. (**A**) Localizations of FAs by RIM on a cardiac fibroblast. FAs appear as low-intensity, elongated spots distributed mainly throughout the lamellipodium (inside FA arrow). In contrast, membrane regions without close proximity to the extracellular matrix appeared as brighter areas in RIM images (outside FA arrow). Scale bar = 5 µm. Fluorescent and RIM images of cardiac fibroblasts after incorporation of (**B**) BFL-SM, (**C**) BFL-GM1, (**D**) BFL-PC, (**E**) TopChol, and (**F**) DiIC_18_(7). For full names of compounds see materials and methods. Lipid distributions, nucleus staining, and DiR localization are shown in green, blue, and red, respectively, while RIM images are presented in gray scale. Scale bars, 20 µm.

Figure 2 shows typical distributions of the intercalated reporter molecules 10 min after delivery. For example, BFL-SM was homogenously distributed within the lamellipodium with small, point-like intracellular signals, larger, unfused liposomes on top of the plasma membrane, and accumulations around the nucleus (Figure 2B). The cellular distribution of BFL-GM1 was similar to that of BFL-SM, except for the missing strong signals close to the nucleus (Figure 2C). In contrast to all other applied fluorescent lipids, here, a strong background signal appeared with time. This could be traced to BFL-GM1 molecules freely diffusing within the cell culture medium, presumably complexed by some compound of fetal bovine serum (see supporting material, Figure S3). The phospholipid derivate BFL-PC showed an inhomogeneous membrane distribution almost throughout the whole cell body except for the outer lamellipodial regions where diffusion analysis was carried out (Figure 2D). The cholesterol derivate TopChol was homogenously distributed mainly in the lamellipodium but also showed strong accumulations around the nucleus (Figure 2E). The small and intense dots identified as cholesterol delivery particles had to be avoided during data collection. In contrast to all other fluorescent tracers, DiIC_18_(7) was evenly distributed almost completely throughout the whole cell body without any enrichment in cellular compartments (Figure 2F).

### 2.3. Diffusion of Fluorescent Lipids in the Plasma Membrane of Cardiac Myofibroblasts

**Figure 3:**
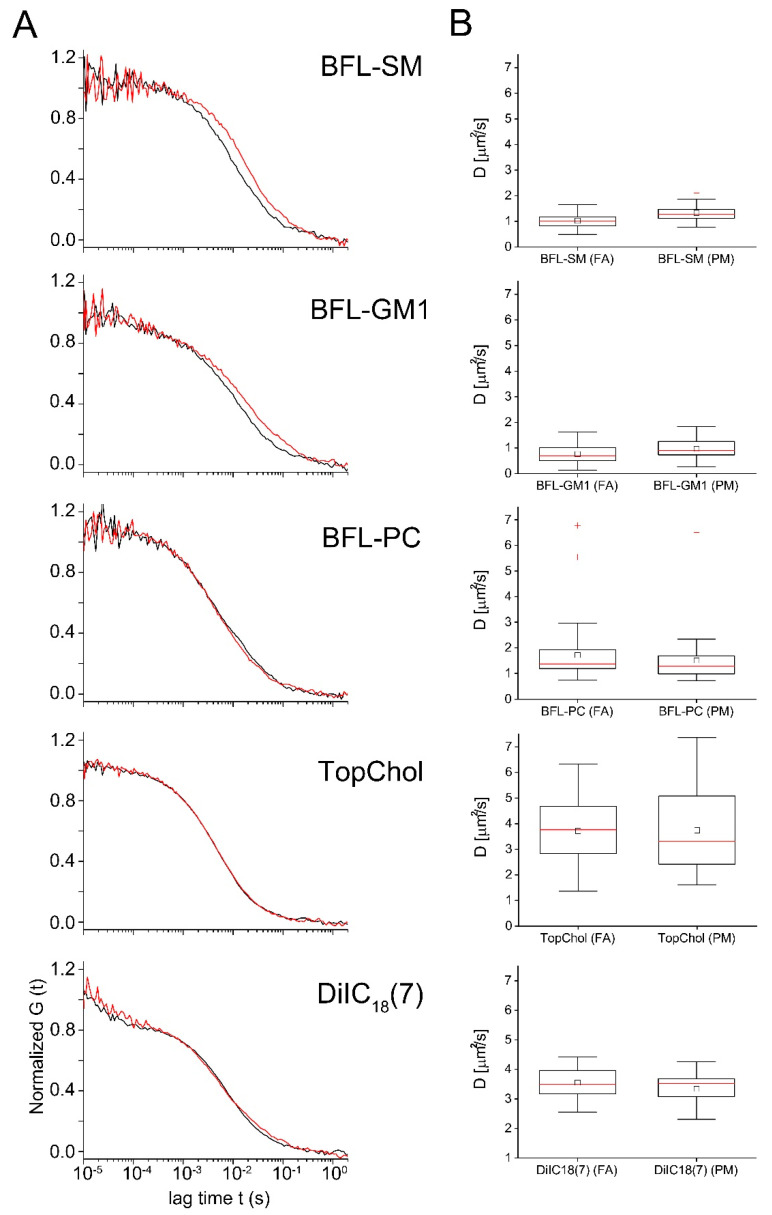
Fluorescence correlation spectroscopy (FCS) measured inside and outside FAs. The indicated fluorescent tracer lipids were incorporated into cells and their diffusion constants measured with FCS. (**A**) Normalized autocorrelation curves inside (red) and outside (black) of FAs. Representative curves are shown. (**B**) Box plots of the resulting diffusion constants. Central small square average, red line median, large box is limited by 75% and 25% quartiles, whiskers extreme values of data, and red crosses show outliers (values whose distance to the interquartile box exceeds 1.5 times the box height). Fit results, statistics, and significance levels can be found in Table 1.

Fluorescent correlation spectroscopy was used to determine the diffusion characteristics of intercalated molecules inside and outside of FAs. All measured autocorrelation functions were fitted assuming a one component, two-dimensional diffusion model (Equation (1) with f = 0), the only exemption being BFL-GM1, where a noticeable transfer of fluorescent molecules into the medium (cf. Supplementary Material Figure S3) necessitated the use of a two-component model. In all other cases, the use of a two-component model destabilized the fit procedure or resulted in one component compensating artefacts at very large or very short lag times. Examples for fit quality are shown in Supplementary Material Figure S4. Fit results are summarized in Figure 3 and in Table 1.

We first describe the results for microdomain-associated lipids. BFL-SM exhibited a significantly reduced diffusion constant inside focal adhesions (average 1.02 µm^2^/s; standard deviation (sd) 0.28 µm^2^/s) as compared to outside (1.33 µm^2^/s; sd 0.29 µm^2^/s). Particle number analysis indicated no enrichment of BFL-SM inside FAs (Table 1). Fluorescence bursts during single FCS measurements, however, were significantly higher throughout these membrane areas raising the possibility of an inhomogeneous molecular distribution.

The other microdomain-associated membrane lipid, BFL-GM1, had to be analyzed by a two-component model (see above). We found that the diffusion of the slower species representing membrane bound lipids (see Supplementary Material) was even slower than that of BFL-SM (Figure 3). Moreover, it was also slower inside FAs (0.76 µm^2^/s; sd 0.35 µm^2^/s) than outside (0.97 µm^2^/s; sd 0.4 µm^2^/s) without molecular enrichment or depletion (Table 1). Additionally, prominent burst phases were detected in both PM regions with especially high values inside FAs.

The diffusion behavior of fluorescently labeled cholesterol (TopChol), the smallest microdomain associated membrane component, significantly differed from that of SM and GM1. Independent of FAs TopChol diffused within the plasma membrane with the same high diffusion constant of about 3.7 µm^2^/s. Similar high diffusion constants were only measured in case of the synthetic membrane probe DiIC_18_(7) (3.4 µm^2^/s; sd 0.7 µm^2^/s), which was not involved in microdomain formation. Both molecules were homogenously distributed inside and outside of the FAs, as indicated by the nearly identical particle numbers detected in the focal volumes (Table 1).

The fluorescently labeled phospholipid component, BFL-PC, neither showed significantly different diffusion constants inside (1.51 µm^2^/s; sd 0.9 µm^2^/s) and outside (1.72 µm^2^/s; sd 1.1 µm^2^/s) FAs nor any enrichment in these membrane regions (Figure 3 and Table 1).

### 2.4. Influence of Lipid Order on the Lipid Diffusion in Giant Unilamellar Vesicles (GUVs)

**Figure 4:**
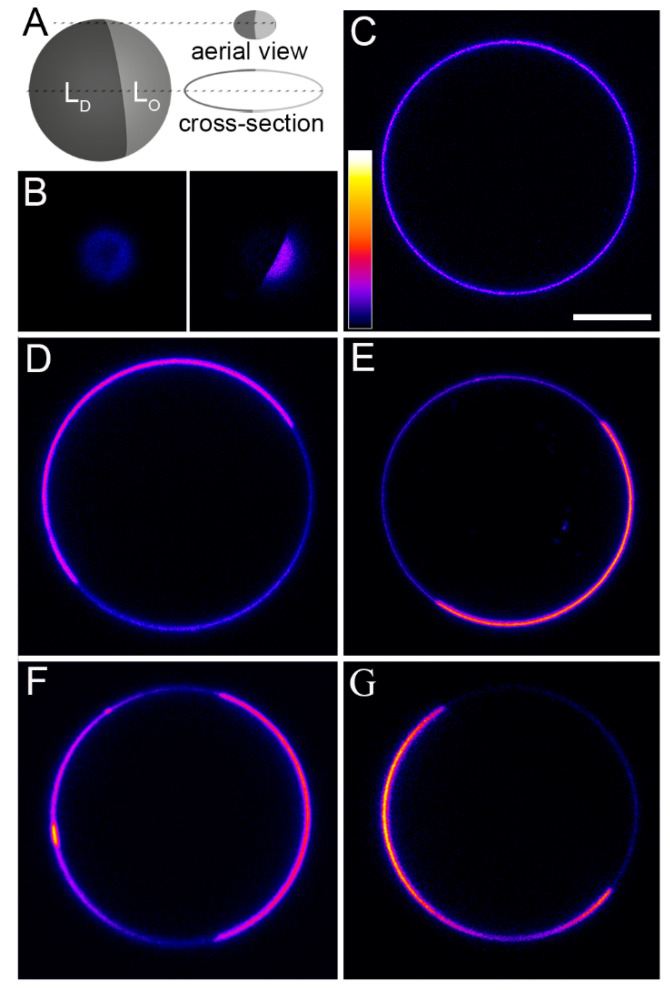
L_o_/L_d_-phase separated giant unilamellar vesicles (GUVs) containing different fluorescent membrane tracers. FCS measurements were performed at the top of the vesicle (see aerial view in (**A**)). Only vesicles phase separation into two large domains both visible at the apex as in (**A**), side view, were analyzed. (**B**) Aerial view of GUV: BFL-SM (left image) showed no clear enrichment in either the L_o_ or L_d_-phase, thus DiIC_18_(7) (right image) served as a clear marker to distinguish both phases. (**C**) Cross section of the same GUV where DiIC_18_(7) is clearly enriched in one phase (see (**B**)), whereas BFL-SM is homogenously distributed. (**D**) BFL-GM1 is slightly enriched in the L_d_-phase, as identified by DiIC_18_(7) (not shown). (**E**) BFL-PC is also enriched in the L_d_-phase, as well as (**F**) TopChol. (**G**) DiIC_18_(7) is almost completely separated into the L_d_-phase, thus no FCS measurements could be performed in the L_o_-phase. Color bar represents the lowest (black—0) and the highest fluorescence intensities (white—255), fire lookup table of Fiji. Scale bar, 10 µm.

To elucidate the influence of lipid order on diffusion within the plasma membrane, diffusivities were also measured in a model membrane system containing membrane domains with more and less order in the hydrophobic acyl chain region of the lipid bilayer (L_o_—liquid ordered phase and L_d_—liquid disordered phase, respectively). Giant unilamellar vesicles (GUVs) were prepared with a lipid composition of SM/DOPC/cholesterol (1/1/1 mol/mol), well known for the simultaneous presence of membrane domains with L_o_ and L_d_ phases [20]. The latter was visualized and identified using the membrane dye DiIC_18_(7) that was entirely excluded from the L_o_ phase. Sphingomyelin exhibited no preference for either phase, all other fluorescent membrane components were distributed in both phases with more or less enrichment in the L_d_ phase, as shown in Figure 4.

FCS measurements were performed at the top of the vesicle (see aerial view in Figure 4A), where both lipid phases were present and possible membrane interactions with the cover glass could be excluded. Only vesicles presenting clear-cut phase separation comparable to the sketch in Figure 4A, side view, were analyzed. To avoid drift, vesicles were bound to the avidin coated cover glass by biotin tethered to their membranes.

**Figure 5:**
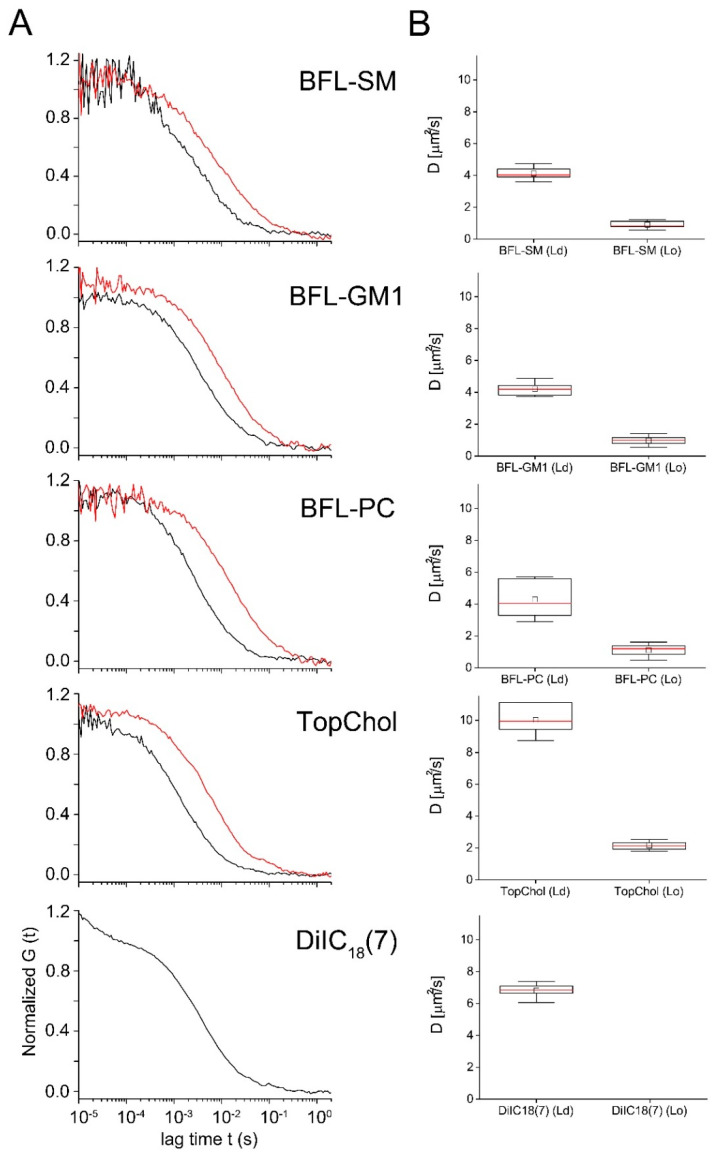
FCS measurements in liquid ordered (L_o_) and liquid disordered (L_d_) domains. The indicated fluorescent lipid tracers were incorporated into GUVs that underwent L_o_/L_d_ phase separation (cf. Figure 4). (**A**) Normalized autocorrelation curves of representative measurements in L_d_-phases (black) and L_o_-phases (red). (**B**) Box plots of the resulting diffusion constants. Central small square average, red line median, large box limited by 75% and 25% quartiles, and whiskers extreme values of data. DiIC18(7) was completely excluded from the Lo phase. Fit results, statistics, and significance levels can be found in Table 2.

All molecules that could be analyzed in both phases (that is, all besides DiIC_18_(7)) clearly showed a much slower diffusion in the liquid ordered phase. Autocorrelation functions are shown in Figure 5. Within each phase, diffusion constants were similar for BFL-SM, BFL-GM1, and BFL-PC with values around 4.2 µm^2^/s in the L_d_ and 1.0 µm^2^/s in the L_o_ phase (for exact values see Table 2). TopChol diffused more than twice as fast as the phospholipids and microdomain-associated lipids in both phases of the GUVs. DiIC_18_(7) diffused faster in the disordered phase than molecules of similar or higher molecular weight such as BFL-SM or BFL-PC (Figure 5 and Table 2). The fluorescent phospholipid BFL-PC and the cholesterol derivate TopChol were enriched in the L_d_ phase as indicated both by fluorescence microscopy (Figure 4) and particle number analysis with FCS (Table 2), whereas BFL-SM showed no preference for either phase. For BFL-GM1 fluorescence microscopy indicated a small preference for the L_d_ phase (Figure 4D), but particle analyses in FCS resulted in differences of no statistical significance (Table 2). All molecules were homogenously distributed within the respective lipid phases, as shown by the small variances of diffusion constants determined for individual FCS measurements (Table 2).

## 3. Discussion

In this study, the lateral diffusion of membrane lipids and lipid analogues have been investigated inside and outside of focal adhesions of cardiac myofibroblasts and within the lipid bilayer of phase separated giant unilamellar vesicles. We tested the hypothesis that transmembrane proteins within FAs alter the lipid order and lead to microdomains of a preferred phase (L_o_ phase or L_d_ phase) [31,32,33,34]. To this end, fluorescently labeled lipid molecules, whose unlabeled versions are generally regarded as microdomain-associated lipids such as sphingomyelin (BFL-SM), GM1 (BFL-GM1), the microdomain excluded lipid phosphatidylcholine (BFL-PC) and fluorescent cholesterol (TopChol), and a synthetic membrane probe, the carbocyanine dye (DiIC_18_(7)), were intercalated into cardiac myofibroblasts using fusogenic liposomes [47]. With this technique fluorescent components are quickly and directly intercalated into the plasma membrane of living cells. This procedure completely bypasses the endosomal pathway and thus substantially minimizes the risk of molecular modification or degradation. Moreover, cell stress was minimized by delivery of small amounts of molecules only.

This intercalation method did not influence the molecular distribution of fluorescent lipids after intercalation. All compounds were found in those cellular compartments that are well known as their native accumulation sites. BFL-SM, BFL-PC, and TopChol were homogenously distributed within the plasma membrane and simultaneously enriched in the ER and the Golgi apparatus [48,49,50]. Additionally, TopChol was also partitioned in small cholesterol delivery compartments called endocytic recycling compartments, as described by Hao et al. [51]. The localization of BFL-GM1 was more complex, because this molecule was cleared relatively quickly from the plasma membrane by transport vesicles (cf. Figure 2C) as well as by solubilization into the cell culture medium (cf. Supplementary Material). Accordingly, its fluorescent signal decreased with time, which hampered its detection as well as FCS measurements. Still, a similar plasma membrane localization was also found by another group [52]. Only the synthetic membrane dye DiIC_18_(7) was not transported to organelles. The most likely reason is that this unnatural molecule was not recognized by any cellular mechanism. It remained homogenously distributed in the plasma membrane for a relatively long time and served as a membrane marker. For molecular distributions, see Figure 2.

Based on the native-like distribution of all used fluorescently labeled lipids, we assumed that their behavior within cells faithfully mimicked that of their unlabeled counterparts. Diffusion constants were determined by FCS. This technique analyzes the motion of fluorescent molecules into and out of the focal spot of the confocal microscope. Thus, the values obtained are averaged over the respective length scale (500 nm diameter for excitation at 488 nm and 600 nm for 633 nm, respectively). As this cell type exhibits FAs that are larger than this length scale, FCS can distinguish between inside and outside of FAs. Nevertheless, Patla et al. [53] examined FAs with cryo-electron tomography and found a pronounced nanostructure. Obviously, diffusion constants measured with FCS cannot resolve these substructures.

Using this technique, we found significantly slower diffusion for the microdomain associated lipids SM and GM1 inside focal adhesions as compared to membrane regions close by. Moreover, we found a prominent spread of single measurements, especially inside FAs. However, particle number analysis indicated no enrichment of these molecules within FAs. This result was surprising because Gaus et al. [31] have reported enhanced lipid order in these areas. Equally surprising was the fact that the diffusivity of BFL-PC and TopChol did not depend on the presence of FAs. In other words, we observed a significant reduction in diffusion within the FA region for SM and also for GM1 but not for other fluorescent molecules. Please note that the distinctly different behavior of the different labeled lipids also excludes artefacts due to membrane geometry, such as the finite size of FAs or the curving away from the substrate at the outer rim of FAs that was also observed by Patla et al. [53] and Medalia et al. [54]. Such effects would influence the diffusion of all membrane molecules in exactly the same way.

To clarify the situation diffusion of all labeled lipids was also analyzed in model giant unilamellar vesicles (GUVs) containing two different liquid crystalline phases, one with more order in the hydrophobic lipid chain region mimicking the lipid ordered phase of membrane microdomains (L_o_) and another with less order similar to the lipid disordered phase outside of the microdomains (L_d_) [55]. These experiments also served to clarify the phase preferences of the fluorescently labeled molecules.

First, we have to discuss the peculiar behavior of TopChol. As fluorescent cholesterol, in contrast to its unlabeled counterpart or sphingomyelin and GM1, is not typically found in membrane microdomains [56], we expected differences between their diffusion behaviors. Measurements of cholesterol in GUVs showed homogenous distribution within both lipid phases (L_o_ and L_d_), significantly faster diffusion in the L_d_ phase (10 µm^2^/s) than in the L_o_ phase (2.2 µm^2^/s), and enrichment in the less ordered L_d_ phase. This is supported by studies of fluorescent cholesterol derivatives with 6-((N-(7-nitrobenz-2-oxa-1,3-diazol-4-yl)amino)hexanoyl (NBD) and fluorescein isothiocyanate (FITC) labeling [42,43], however one study with a newly synthesized BODIPY cholesterol analog reported the opposite behavior [57]. In living cardiac myofibroblasts, TopChol distribution appeared as expected for cholesterol. However, we measured surprisingly fast diffusion inside as well as outside of FAs (3.7 µm^2^/s). As this value is more than twice as large as the diffusion constants of any other natural lipids tested and only the fully artificial DiIC_18_(7) displayed similar fast diffusion in cell membranes, we see a clear difference to the reported behavior of unlabeled cholesterol. Overall, the behavior of TopChol and DiIC_18_(7) was comparable in model membranes and in cellular membranes. Both molecules enriched in the L_d_ phase, TopChol partially, DiIC_18_(7) exclusively, and diffused unusually fast in the lipid bilayer (10 µm^2^/s and 6.8 µm^2^/s). This at least two times faster diffusion also suggests a lack of molecular interaction with other membrane lipids. For closely related BODIPY-modified cholesterols, Solanko and coworkers also reported unexpectedly high diffusion constants from which they concluded that the attached fluorescent labels hindered cholesterol interactions with membrane lipids [57]. Thus, it appears that TopChol behaves in some respects as unphysiological as the completely artificial alkyl-carbocyanine membrane labels.

In model membranes undergoing L_d_/L_o_ phase separation, fluorescently labeled lipids usually prefer one of the two phases [42]. However, in our experiments SM and GM1 exhibited no preference for either phase, while BFL-PC, TopChol, and DiIC18(7) clearly preferred the L_d_ phase. Nevertheless, SM and GM1 diffused about four times faster in the less ordered L_d_ domains. This diffusion behavior significantly differed from that within the plasma membrane where the difference amounted to only about 30%. Even more striking is the same comparison for the fluorescent PC analog. While in model membrane diffusion in the less ordered L_d_ phase was also four times faster than in L_o_, no significant difference was found between plasma membrane regions within FAs and next to them.

Hence, by comparison of diffusion constants between L_d_/L_o_ phases in model systems, the partial change in diffusion constants in and outside of FAs in the cell membrane and the lack of molecular enrichment in FAs, we conclude that there is no clear signature of classical liquid ordered or liquid disordered phases in FAs. Instead, some membrane lipids, which are slowed down within FAs are more likely to interact with molecular constituent(s) of FAs. This conclusion is in line with the findings of Eggeling and co-workers, who also postulated alternative interactions responsible for the strong local trapping of a sphingolipid analogue in the basal membrane of epithelial kidney cells instead of the presence of nanodomains [58].

## 4. Materials and Methods

### 4.1. Cell Culture

Cardiac fibroblasts and myocytes were isolated as described in [46] from 18 day old Wistar rat embryos, yielding fibroblasts and myocytes in an approximately 2:1 ratio. After isolation, 5 × 10^5^ cells were seeded on cell culture dishes (6 cm diameter, Nunc, Wiesbaden, Germany) and maintained in F10 Ham’s medium (Sigma-Aldrich, St. Louis, MO, USA) supplemented with 10% fetal bovine serum (Biochrom, Berlin, Germany), a 1:100 dilution of an antibiotic solution (10,000 units penicillin and 10 mg/mL streptomycin in 0.9% NaCl, Sigma-Aldrich, Taufkirchen, Germany)), and a 1:200 dilution of solution containing insulin (1 mg/mL), transferrin (0.55 mg/mL), and sodium selenite (0.5 µg/mL) (Sigma-Aldrich) at 37 °C and 5% CO_2_ in saturated humid atmosphere. The next day, dishes were gently washed with phosphate buffered saline (PBS; 137 mM NaCl, 8.1 mM Na_2_HPO_4_, 2.68 mM KCl, 1.47 mM KH_2_PO_4_, pH 7.4) to remove cellular debris and non-adherent cells. A 5 day proliferation phase, where medium was exchanged every two days, resulted in differentiated myofibroblasts with prominent supermature FAs and very low levels of cell division. Two days before the experiment, cells were trypsinated, and 10,000 cells were seeded on microscope culture dishes that were prepared as follows.

Thickness-corrected high precision cover glasses (170 ± 5 µm, 22 × 22 mm; Marienfeld, Lauda-Königshofen, Germany) were plasma cleaned (Diener electronics, Ebhausen, Germany) for 2 min and glued over 1.8 cm holes in 3 cm culture dishes using polydimethylsiloxane (Sylgard184, Dow Corning Co., MI, USA). Human plasma fibronectin (BD Biosciences, San Jose, CA, USA) was used at a concentration of 10 µg/mL in PBS for coating (30 min at 37 °C).

### 4.2. Identification of Myocytes and Fibroblasts by Immunostaining and Phase Contrast Microscopy

Primary rat embryonic myofibroblasts were incubated first in paraformaldehyde solution (3.7%) (MerckMillipore, Darmstadt, Germany) in cytoskeletal buffer (C–150 mM NaCl; 5 mM MgCl_2_; 5 mM ethylene glycol-bis(2-aminoethylether)-N,N,N’,N’-tetraacetic acid (EGTA); 5 mM glucose; 10 mM 2-(4-morpholino)ethanesulfonic acid (MES); pH 6.1) for 30 min at 37 °C. Subsequently, cells were washed with 30 mM glycine-CB solution and gently permeabilized using Triton-X100 (0.5%) in CB buffer for 10 min.

Unspecific labelling was reduced by incubating cells with blocking solution (5% solution of dry milk (Carl Roth, Karlsruhe, Germany) in CB) for 90 min. Cells were then incubated with a 1:100 dilution (in 1% solution of dry milk in CB) of primary antibodies, mouse sarcomeric anti-α-actinin, clone EA-53 monoclonal antibody (A7811, Sigma-Aldrich) overnight at 4 °C. Cy3-conjugated goat anti-mouse (115–165-006, Jackson Immunoresearch, Baltimore Pike, PA, USA) secondary antibody was used at 1:200 dilution for fluorescent labeling. For F-actin visualization, CytoPainter Phalloidin-iFluor488 (AB176753, Abcam, Cambridge, UK) was added to the cells at 1:300 dilution in parallel to the secondary antibody and incubated at RT for 2 h. Subsequent staining of cell nuclei was carried out using the specific nucleus staining reagent NucBlue (R37605, Molecular Probes, Eugene, OR) following the manufacturer’s guidelines. After washing three times with CB, samples were stored in the same buffer and analyzed by phase contrast and fluorescence microscopy.

### 4.3. Transfection of Myofibroblasts with GFP-Vinculin

To identify FAs by RIM, the above described myofibroblasts and myocytes were transfected using GFP-vinculin [59] by electroporation and the fluorescence signal of GFP-vinculin was compared with the interference pattern of adhered cells. For plasmid delivery, primary rat cardiomyocytes nucleofector kit (Lonza, Köln, Germany) was used whereby ca. 500,000 cells were electroporated (Lonza, Köln, Germany) with 5 µg plasmid dissolved in 82 µl nucleofection solution and 18 µl supplement. After, poration cells were resuspended in fresh medium and seeded on glass surfaces and analyzed 24 h later by microscopy.

### 4.4. Lipids

The lipids 1,2-Dioleoyl-*sn*-glycero-3-phosphoethanolamine (DOPE), 1,2-dioleoyl-3-trimethylammonium-propane, chloride salt (DOTAP), 23-(dipyrrometheneboron difluoride)-24-norcholesterol (TopChol), 1,2-dioleoyl-*sn*-glycero-3-phosphocholine (DOPC), *N*-palmitoyl-d-*erythro*-sphingosylphosphorylcholine (SM), cholesterol, and 1,2-dihexadecyl-*sn*-glycero-3-phosphoethanolamine-N-(cap biotinyl) (CapBio-DHPE) were purchased from Avanti Polar Lipids (Alabaster, AL, USA). The lipids 1,1’-dioctadecyl-3,3,3’,3’-tetramethylindotricarbocyanine iodide (DiIC_18_(7)), 2-(4,4-difluoro-5,7-dimethyl-4-bora-3*a*,4*a*-diaza-*s*-indacene-3-dodecanoyl)-1-hexadecanoyl-*sn*-glycero-3-phosphocholine (BFL-PC), *N*-(4,4-difluoro-5,7-dimethyl-4-bora-3a,4a-diaza-*s*-indacene-3-dodecanoyl)sphingosyl phosphocholine (BFL-SM), and *N*-(4,4-difluoro-5,7-dimethyl-4-bora-3a,4a-diaza-*s*-indacene-3-pentanoyl)ganglioside (BFL-GM1) were ordered by Life Technologies (Eugene, OR, USA).

### 4.5. Cell Staining Using Fusogenic Liposomes

To stain adherent cells, fusogenic liposomes (FLs) were used. Liposomes were produced as described in [47]. Briefly, DOPE, DOTAP, and fluorescently labeled lipid or membrane tracer molecules were mixed in chloroform at a weight ratio of DOPE/DOTAP/fluorescent dye of 1/1/0.05–0.1. Chloroform was evaporated in vacuum and the dried lipids were immediately dispersed in 20 mM HEPES buffer (pH 7.4; VWR, Darmstadt, Germany), at a total lipid concentration of 2 mg/mL. The solution was vortexed for 1–2 min and subsequently sonicated for 10 min. Before cell staining, FLs were diluted 1:100 in F10 Ham’s medium without supplements. Adherent cells were washed twice with warm PBS, the staining solution was applied at room temperature, and samples were placed at 37 °C. Fusion efficiencies between 50% and 90% of stained adherent cells were achieved regularly after 10 min incubation. The culture dish was washed again to remove unfused liposomes and refilled with warm myocyte growth medium.

### 4.6. Giant Unilamellar Vesicles (GUVs)

L_d_/L_o_ phase separated GUVs were produced from ternary mixtures of DOPC, SM, and cholesterol at a molar ratio of 1/1/1. Fluorescent lipids were added to this basic lipid mixture at 0.1 mol% for confocal imaging and at 0.002 mol% for FCS measurements. Furthermore, CapBiotin-DHPE was added at 0.3 mol%. Vesicles were prepared via electroswelling [60]. To this end, 100 µl of the lipids dissolved in chloroform (1 mg/mL) were dispensed on two glass slides coated with indium tin oxide and chloroform was evaporated in a vacuum chamber for 1 h. The lipid coated slides were then mounted in a Teflon frame providing a 1 mm gap. The Teflon swelling chamber was heated to 48 °C and warm sucrose solution (280 mOsm/L) was added. An alternating voltage of 1.4 V and 10 Hz was applied for 1 h, which led to formation of GUVs. GUVs were slowly cooled down to room temperature overnight and transferred to the observation chamber.

This chamber consisted of an upper and lower polypropylene part with a cover glass glued to the lower one. Cover glasses were coated with neutravidin (NAV; 0.5 mg/mL in PBS for 1 h followed by five times rinsing; Life Technologies, Eugene, OR, USA) before GUV addition. The chamber was then completely filled with 1.9 mL of glucose solution (280 mOsm/L) and 100 µl of GUVs. A silicone gasket was put between the upper and lower part and the chamber was screwed together, thus forming an inner pool of 2 mL volume. This prevented evaporation and changes in buffer osmolarity during FCS measurements. GUVs were left to adhere to the cover glass for 1 h before measurements.

### 4.7. Supported Lipid Bilayer

Supported lipid bilayers (SLB) were prepared using a film balance (Nima, Coventry, UK) applying Langmuir-Blodgett-Langmuir-Schäfer technique. The subphase was ultrapure water. Both membrane layers, the proximal layer on the glass surface as well as the distal layer facing the buffer, were formed by DPPC and BFL-GM1 at a molar ratio of 1/0.002. Transfer pressure was set to 20 mN/m. SLBs were kept under PBS or PBS completed with 10% bovine serum albumin (BSA; Biochrom, Berlin, Germany) and were used immediately after preparation (for results see Figure S3c).

### 4.8. Confocal Microscopy and Fluorescence Correlation Spectroscopy (FCS)

Confocal microscopy and FCS measurements were done using a confocal laser scanning microscope (LSM 710; Carl Zeiss MicroImaging GmbH, Jena, Germany) equipped with an argon ion laser (488 nm), a red helium-neon laser (633 nm), and accessories for FCS measurements (ConfoCor 3 and software autocorrelator, Carl Zeiss). A 40xC-Apochromat water immersion objective (Carl Zeiss) with a numerical aperture of 1.2 was used. Fluorescence of Bodipy FL (ex. 488 nm) was detected in the spectral region of 500–550 nm for confocal microscopy and 515–530 nm for FCS measurements. DiIC_18_(7) (ex. 633 nm) was detected with a long pass filter LP 650 nm (confocal images and FCS). Each FCS measurement consisted of 6–10 repeats with a 3 s pre-bleach period. Laser intensities were always kept below 0.1 mW as suggested for intracellular FCS measurements [61]. Measured autocorrelation functions *G*(*t*) were fitted by the two dimensional diffusion model of Schwille [62] extended to two diffusing species using the manufacturers software.

(1) $$G\left(t\right)=\frac{1}{N}\frac{1-T\left(1-\exp\left(-\frac{t}{\tau_{trip}}\right)\right)}{1-T}\left(\frac{f_{}}{1+\frac{t}{\tau_{D_{1}}}}+\frac{1-f_{}}{1+\frac{t}{\tau_{D_{2}}}}\right)$$

Here, *t* denotes the correlation time (often also called lag time), *N* is the number of independent particles in the focal volume, *T* is the triplet fraction, τ_trip_ is the lifetime of the triplet state, τ_D_ is the diffusion time given by $\tau_{D}=\frac{\omega^{2}}{4D}$ with ω the radius of the point spread function (determined with fluorescent beads to be 251 nm for the argon ion laser and 302 nm for the HeNe laser), and D is the diffusion constant. $f_{}$ is the percentage of molecules of species 1, which in case of the 1-component fit, is set to 1 and, in case of the 2-component fit, used as a fit parameter.

RIM images were obtained using the argon ion laser (514 nm) passing through a T80/R20 main beam splitter. For FCS analysis on cells only clearly identifiable focal adhesions were used. We restricted our analyses to the lamellipodial region where the cell was very thin, and the probability of intracellular fluorescent particles disturbing the measurement was accordingly low.

### 4.9. Statistical Analyses

Statistical analyses of data were performed in MATLAB (R2020, MathWorks, Natick, MA, USA) using the T-test and the non-parametric Wilcoxon rank sum test. Statistical significance was indicated as follows: * *p* ≤ 0.05, ** *p* ≤ 0.01, *** *p* ≤ 0.001. Data are expressed as mean and standard deviation (sd).

## 5. Conclusions

Here, we analyzed the diffusion of membrane lipids inside and outside of focal adhesion complexes of living cells to infer on their molecular interplay with other membrane components. We found significantly reduced diffusion constants (D) for the microdomain associated lipids SM and GM1 inside focal adhesions, yet without enrichments in these membrane parts. Diffusion of other phospholipids or synthetic lipid analogues did not depend on FAs. However, in model membranes, all analyzed lipids diffused much faster within the L_d_ phase than within L_o_. Therefore, we postulate that protein–lipid interactions and not microdomain formation are slowing down SM and GM1 molecules in FAs.

## Figures and Tables

**Table 1:** Characteristic diffusion parameters of different fluorescent membrane tracers determined by FCS inside (FA) and outside (PM) of FAs of cardiac myofibroblasts.

	D (μm^2^/s)	sd_D_ (µm^2^/s)	Signi. D		N_Part_	sd_Part_	Signi. N_Part_		N_meas_
			T-Test	Rank Sum Test			T-Test	Rank Sum Test	
BFL-SM (FA)	1.0	0.3	***	***	23	17	n.s.	n.s.	54
BFL-SM (PM)	1.3	0.3			21	17			54
BFL-GM1 (FA)	0.8	0.3	***	*	38	30	n.s.	n.s.	37
BFL-GM1 (PM)	1.0	0.4			41	54			37
BFL-PC (FA)	1.5	0.9	n.s.	n.s.	21	14	n.s.	n.s.	45
BFL-PC (PM)	1.7	1.1			27	18			45
TopChol (FA)	3.7	1.4	n.s.	n.s.	29	15	n.s.	n.s.	35
TopChol (PM)	3.7	1.6			32	18			35
DiIC_18_(7) (FA)	3.1	0.4	n.s.	n.s.	18	15	n.s.	n.s.	17
DiIC_18_(7) (PM)	3.7	0.7			25	20			16

D: diffusion constant; N_Part_: number of independently diffusing particles within the confocal volume; sd: standard deviations of the respective parameters; signi: statistical significance; N_meas_: number of different cells probed; n.s.: non-significant, * *p* ≤ 0.05, *** *p* ≤ 0.001.

**Table 2:** Characteristic diffusion parameters of different fluorescent membrane tracers determined by FCS in the liquid ordered (L_o_) and liquid disordered (L_d_) phases of GUVs.

	D (μm^2^/s)	sd_D_ (µm^2^/s)	Signi. D		N_Part_	sd_Part_	Signi. N_Part_		N_meas_
			T-test	Rank Sum Test			T-test	Rank Sum Test	
BFL-SM (L_o_)	0.9	0.2	***	***	72	59	n.s.	n.s.	10
BFL-SM (L_d_)	4.1	0.4			146	134			8
BFL-GM1 (L_o_)	1.0	0.3	***	***	36	26	n.s.	n.s.	9
BFL-GM1 (L_d_)	4.2	0.4			48	23			10
BFL-PC (L_o_)	1.1	0.3	***	***	12	7	***	***	12
BFL-PC (L_d_)	4.3	1.1			58	38			11
TopChol (L_o_)	2.2	0.2	***	***	34	7	***	**	7
TopChol (L_d_)	10	0.9			62	11			7
DiIC_18_(7) (L_d_)	6.8	0.4	-	-	3	1	-	-	7

D: diffusion constant; N_Part_: number of independently diffusing particles within the confocal volume; sd: standard deviations of the respective parameters; signi: statistical significance; N_meas_: number of different cells probed; n.s.: non-significant, ** *p* ≤ 0.01,*** *p* ≤ 0.001.

## Supplementary Materials

The following are available online at https://www.mdpi.com/1422-0067/21/21/8149/s1, Figure S1: S1 Identification of cardiac myocytes and myofibroblast in primary culture, Figure S2: Fluorescent components incorporated into the PM of cardiac myofibroblasts, Figure S3: Increase of background signal when using BFGl-GM1 in cells and model systems, Figure S4: Diffusion analyses of sphingomyelin in different membrane systems.
