# Supplementary material for: Fluorescence Correlation Spectroscopy Reveals Interaction of Some Microdomain-Associated Lipids with Cellular Focal Adhesion Sites
Source: Int J Mol Sci. 2020 Oct 31;21(21):8149. doi: 10.3390/ijms21218149 (PMC7662714; doi:10.3390/ijms21218149)
Supplement: Supplementary file 1 [file ijms-21-08149-s001.pdf]

Supplementary Information

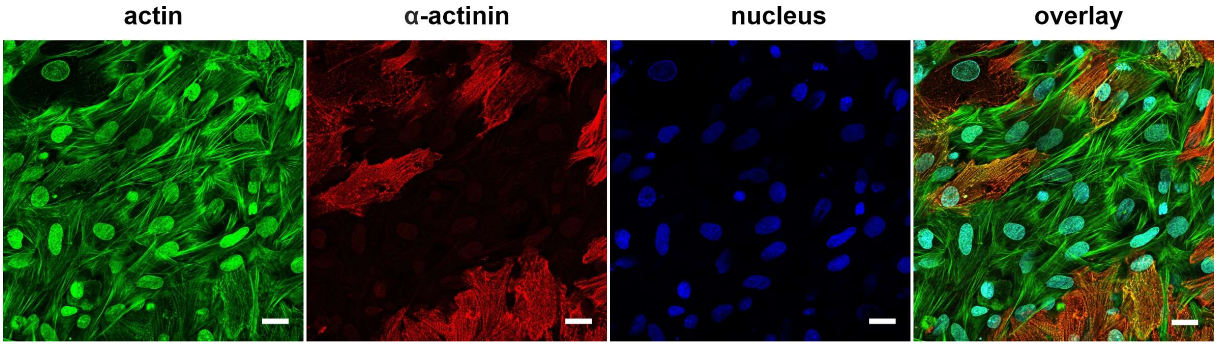

Figure S1 Identification of cardiac myocytes and myofibroblast in primary culture. The two cell types were clearly distinguished based on the immune staining of actin cytoskeleton with Alexa488-phalloidin (green), and the α-actinin staining with anti-α-actinin (red). Cell nuclei were visualized using NucBlue (blue). Myocytes were identified by a co-localized actin-cytoskeleton network and α-actinin (yellow). Scale bar, 20 μm.

A)

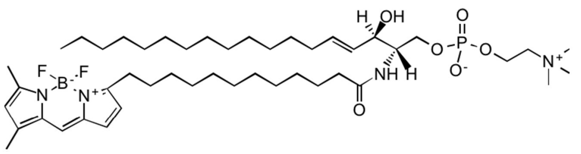

B)

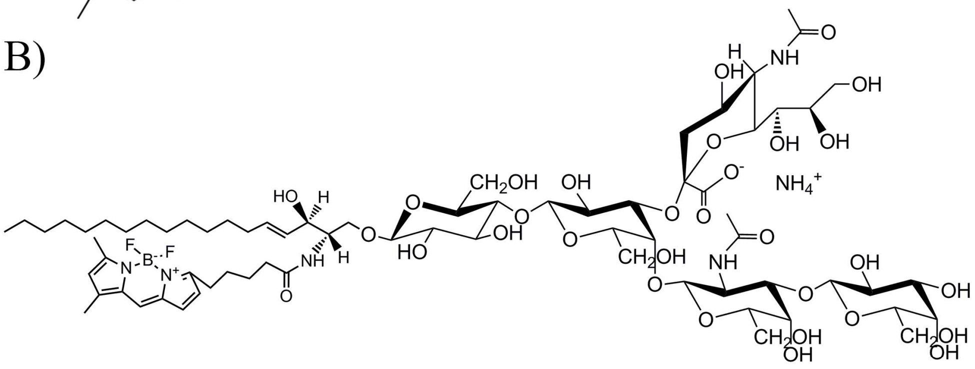

C)

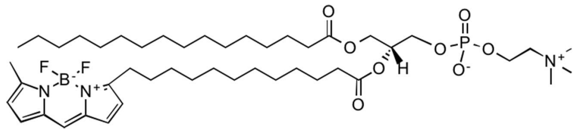

D)

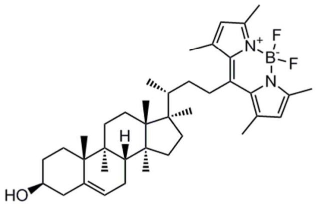

E)

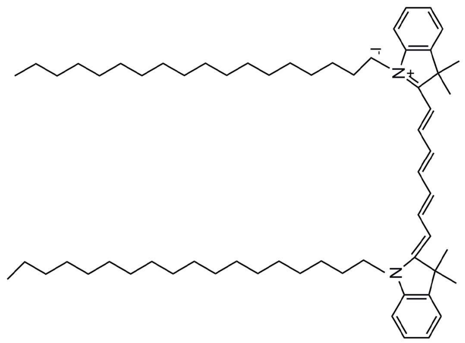

Figure S2 Fluorescent components incorporated into the PM of cardiac myofibroblasts. A) *N*-(4,4-difluoro-5,7-dimethyl-4-bora-3a,4a-diaza-*s*-indacene-3-dodecanoyl)sphingosyl phosphocholine (BFL-SM), B) *N*-(4,4-difluoro-5,7-dimethyl-4-bora-3a,4a-diaza-*s*-indacene-3-pentanoyl)ganglioside (BFL-GM1), C) 2-(4,4-difluoro-5,7-dimethyl-4-bora-3*a*,4*a*-diaza-*s*-indacene-3-dodecanoyl)-1-hexadecanoyl-*sn*-glycero-3-phosphocholine (BFL-PC), D) 23-(dipyrrometheneboron difluoride)-24-norcholesterol (TopChol), and E) 1,1'-dioctadecyl-3,3,3',3'-tetramethylindotricarbocyanine iodide ($DiIC_{18}(7)$).

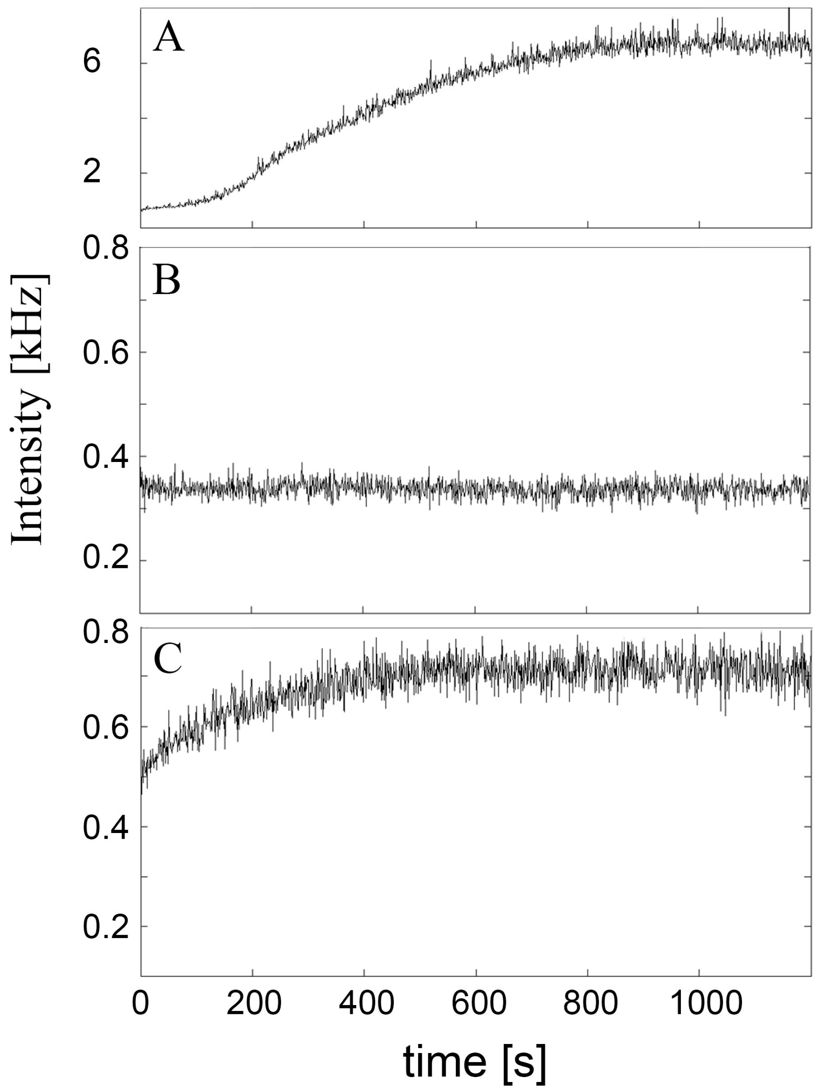

Figure S3 Increase of background signal when using BFGl-GM1 in cells and model systems. The fluorescence intensity was measured 50 µm above A) adherent cells treated with fusogenic vesicles, B) a supported lipid bilayer (DPPC/BFL-GM1 1/0.002 w/w) covered with PBS and C) a supported lipid bilayer (DPPC/BFL-GM1 1/0.002 w/w) covered with PBS + 10% fetal calf serum. Intensities were recorded for 20 min and averaged over 1 µs periods.

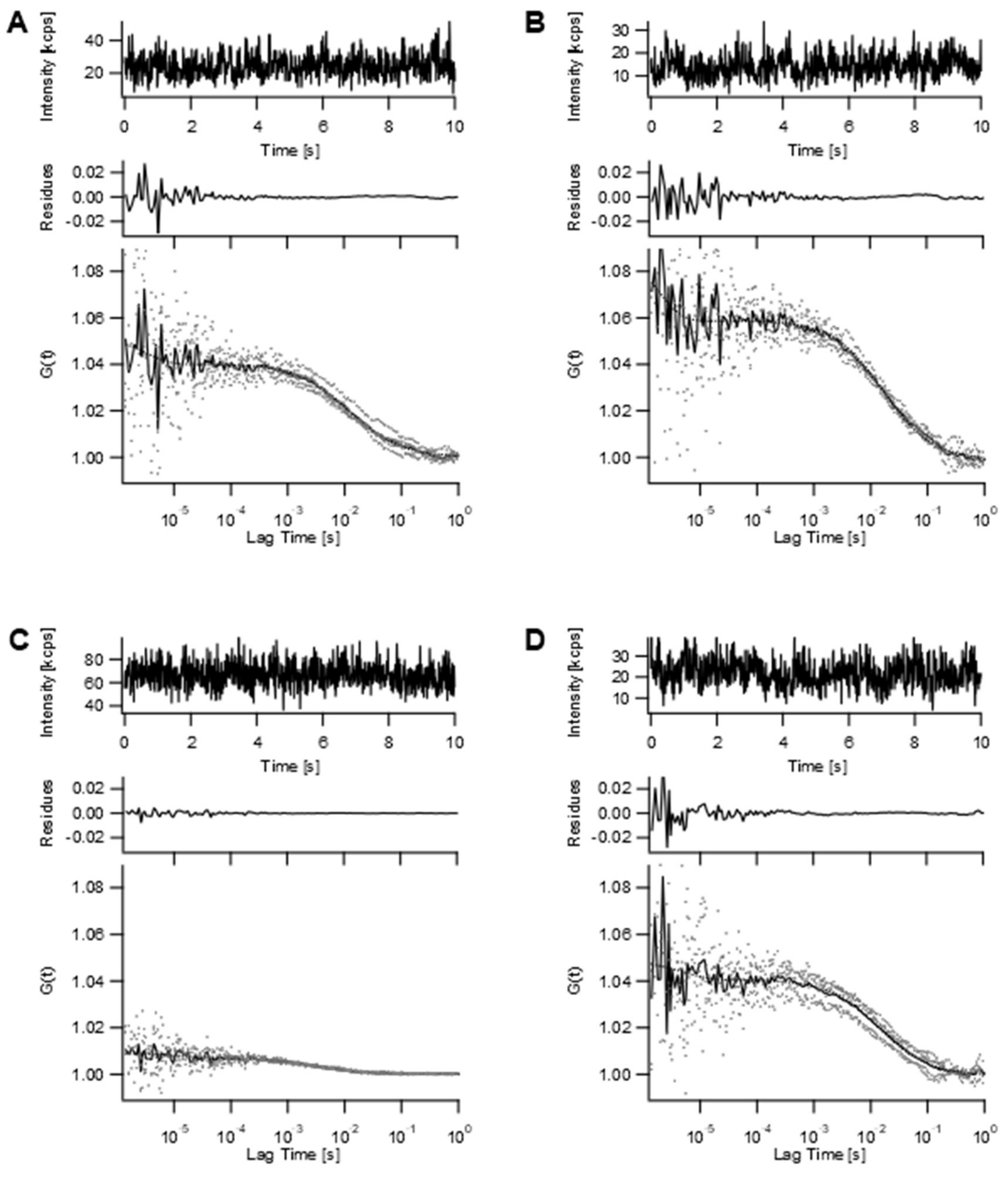

FIGURE S4 Diffusion analyses of sphingomyelin in different membrane systems. Each panel shows (from top to bottom) raw intensity readings in 1 ms intervals, differences between averaged correlogram and fit result, and the correlograms (grey dots: individual data, solid line: averages of repeated measurements, dotted line: result of fit with Eq. 1). A) Free area of the plasma membrane. Fit results in D=1.3 $\mu m^2/s$ and N=25. B) Focal adhesion. Fit results in D=0.97 $\mu m^2/s$ and N=17. C) Liquid disordered phase of a GUV. Fit results in D=5.1 $\mu m^2/s$ and N=144. D) Liquid ordered phase of a GUV. Fit results in D=1.1 $\mu m^2/s$ and N=25. Fits of the individual measurements showed that the noticeable splitting of individual measurements in A and D was due to widely varying contributions of triplett state decay at very short lag times while diffusion constants and particle numbers were consistent in all repeats.
